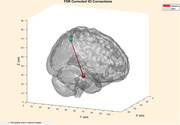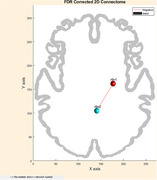# Correlation Between Functional Connectivity in the Default Mode Network (DMN) and Plasma Biomarker Concentrations in Patients with Mild Cognitive Impairment

**DOI:** 10.1002/alz70856_107675

**Published:** 2026-01-09

**Authors:** Gabriela Barbosa Rodrigues, Isadora Cristina Ribeiro, Marjorie Cristina Rocha da SIlva, Liara Rizzi, Brenda Costa Gonçalves, Ítalo Karmann Aventurato, Ananssa Silva, Thaís Lopes Pinheiro, Luis E. Santos, Fernanda Guarino De Felice, Fernando Cendes, Marcio Luiz Figueredo Balthazar

**Affiliations:** ^1^ Universidade Estadual de Campinas ‐ UNICAMP, Campinas, São Paulo, Brazil; ^2^ Universidade Estadual de Campinas (UNICAMP), Campinas, SP, Brazil; ^3^ University of California San Francisco, San Francisco, CA, USA; ^4^ UNICAMP ‐ UNIVERSIDADE ESTADUAL DE CAMPINAS, CAMPINAS, SP, Brazil; ^5^ D'Or Institute for Research and Education, Rio de Janeiro, RJ, Brazil; ^6^ Queen's University, Kingston, ON, Canada; ^7^ Unicamp, Campinas, Brazil; ^8^ University of Campinas (Unicamp), Campinas, SP, Brazil

## Abstract

**Background:**

Disruptions in brain network connectivity are strongly associated with the progression of cognitive decline in the Alzheimer's disease (AD) continuum, including mild cognitive impairment (MCI). This study aimed to investigate the relationship between alterations in functional brain connectivity within the default mode network (DMN) in patients with MCI and plasma biomarker levels typically altered in AD (Aβ40, Aβ42, Tau, pTau‐181, Aβ42/Aβ40, Aβ42/pTau, Aβ42/tTau, pTau/tTau).

**Methods:**

Eighteen patients (mean age = 65 years) diagnosed with MCI according to the 2018 NIA‐AA and Alzheimer's Association criteria, based on medical and neuropsychological evaluation at the Hospital das Clínicas, University of Campinas (HC‐UNICAMP), Brazil, were selected for blood collection and subsequent functional magnetic resonance imaging (fMRI) scans. Plasma samples were stored and analyzed using the automated SIMOA HD‐X immunoassay system (Quanterix, Billerica, MA). Resting‐state fMRI (RS‐fMRI) data were acquired using a 3T Achieva‐Intera PHILIPS® scanner. Both imaging data and correlation analyses were processed using the UF2C toolbox within MATLAB and SPM12, with results corrected for false discovery rate (FDR).

**Results:**

Two notable negative correlations were found between the right hippocampus and right precuneus and the Aβ42/Aβ40 ratio (Spearman's correlation: r = ‐0.76, *p* =  0.033). No significant correlations were observed for other plasma biomarkers after FDR correction.

**Conclusion:**

Since network reorganization is a characteristic feature of MCI and AD, with regions exhibiting increased or decreased activity, the observed inverse relationship between Aβ42/Aβ40 and functional connectivity between the right hippocampus and right precuneus (indicating that an increase in Aβ42/Aβ40 is associated with decreased functional connectivity, and vice versa) supports the disease's underlying pathophysiology. This finding provides a potential avenue for research and diagnostic monitoring. Further studies are needed to explore the functional impact of these alterations and their relevance to disease progression.